# Cytoprotective autophagy as a pro-survival strategy in ART-resistant malaria parasites

**DOI:** 10.1038/s41420-023-01401-5

**Published:** 2023-05-13

**Authors:** Deepika Kannan, Nishant Joshi, Sonal Gupta, Soumya Pati, Souvik Bhattacharjee, Gordon Langsley, Shailja Singh

**Affiliations:** 1 Special Centre for Molecular Medicine, Jawaharlal Nehru University, New Delhi, India; 2 Department of Life Sciences, School of Natural Sciences, Shiv Nadar University, Greater Noida, Uttar Pradesh India; 3 Inserm U1016-CNRS UMR8104, Institut Cochin, Paris, France; 4 Laboratoire de Biologie Comparative des Apicomplexes, Faculté de Médecine, Université Paris Descartes - Sorbonne Paris Cité, Paris, France

**Keywords:** Macroautophagy, Drug discovery

## Abstract

Despite several initiatives to subside the global malaria burden, the spread of artemisinin-resistant parasites poses a big threat to malaria elimination. Mutations in *Pf*Kelch13 are predictive of ART resistance, whose underpinning molecular mechanism remains obscure. Recently, endocytosis and stress response pathways such as the ubiquitin-proteasome machinery have been linked to artemisinin resistance. With *Plasmodium*, however, ambiguity persists regarding a role in ART resistance for another cellular stress defence mechanism called autophagy. Therefore, we investigated whether, in the absence of ART treatment, basal autophagy is augmented in *Pf*K13-R539T mutant ART-resistant parasites and analyzed whether *Pf*K13-R539T endowed mutant parasites with an ability to utilize autophagy as a pro-survival strategy. We report that in the absence of any ART treatment, *Pf*K13-R539T mutant parasites exhibit increased basal autophagy compared to *Pf*K13-WT parasites and respond aggressively through changes in autophagic flux. A clear cytoprotective role of autophagy in parasite resistance mechanism is evident by the observation that a suppression of PI3-Kinase (PI3K) activity (a master autophagy regulator) rendered difficulty in the survival of *Pf*K13-R539T ART-resistant parasites. In conclusion, we now show that higher PI3P levels reported for mutant *Pf*Kelch13 backgrounds led to increased basal autophagy that acts as a pro-survival response to ART treatment. Our results highlight *Pf*PI3K as a druggable target with the potential to re-sensitize ART-resistant parasites and identify autophagy as a pro-survival function that modulates ART-resistant parasite growth.

## Introduction

Maintaining cellular homeostasis primarily involves a balance between the anabolic and catabolic pathways within a cell. Catabolic pathways play a significant role in regulating homeostasis by degrading toxic products such as protein aggregates, damaged organelles, misfolded proteins, or intracellular pathogens. Two key pathways responsible for catabolism are (i) the ubiquitin-proteasome pathway (UPP) and (ii) the autophagy-lysosomal pathway (ALP). UPP is primarily associated with the disposal of small toxic wastes such as misfolded proteins [1, 2], while ALP removes larger toxic wastes such as damaged organelles and protein aggregates [3]. ALP culminates by engulfing damaged material in the cell cytoplasm within membrane-bound vesicles called autophagosomes, followed by fusion with lysosomes to form autolysosomes. Hydrolases within lysosomes break down the cellular material releasing small cargo molecules such as nucleic acids, amino acids, and lipid molecules for reprocessing. Thus, dysregulation of catabolic pathways results in increased stress, proteopathy, and cell death.

In yeast approximately 40 core AuTophaGy-related (Atgs) genes are involved in autophagy [4], and the *Plasmodium* genome encodes several Atgs, indicating the existence and essentiality of parasite ALP [5, 6], where a constitutively active autophagy pathway has been reported for the hepatic and gametocytic stages of the malaria parasites [7–9]. Autophagy responses can be involved in the degradation of developmental stage-specific proteins and organelles to maintain cellular and protein homeostasis [10]. *Plasmodium falciparum* lacks several classical upstream regulatory kinases (such as mTOR orthologues), rendering unclear how autophagy is induced under nutrient limitation and cellular stress conditions. However, a recently identified AMPK homolog (*Pf*KIN) is believed to be the principal determinant for monitoring the nutrient and energy levels and may also contribute to regulating autophagy [11]. Residing in a nutritional environment (red blood cells), *Plasmodium* is also resilient to mild nutrient starvation, however, depletion of growth factors or extended starvation or cellular stress results in hibernation or induction of autophagy [9, 12, 13]. This is consistent with the functional existence of identified downstream proteins of the autophagy pathway in *Plasmodium* [6].

The existence of basal (nutrient-fed) and starvation-induced autophagy in *Plasmodium* has already been illustrated [9, 11, 13, 14]. Mild starvation demonstrates upregulated autophagy with unaltered parasite growth and increased expression of *Pf*Atg5 and *Pf*Atg8 – the autophagosome marker. Howbeit, prolonged starvation manifests as stalled parasite growth and reinvasion, and a decrease in *Pf*Atg5 and *Pf*Atg8 punctae. Concomitantly, prolonged starvation also results in autophagy-induced parasite death, while the parasites retain their ability to withstand mild starvation [9, 13]. This is corroborated with the effect of nutrient deprivation in vivo in *Plasmodium* *berghei* infection [11]. Under nutrient-limited conditions, parasites encountered a significant reduction in the number of merozoites formed per schizont, suggesting transient repression of cell cycle and differentiation, stimulating latency. Several genes involved in the cell cycle, maturation, protein translation, and ion transport were downregulated, while genes associated with signaling (especially kinases) and regulatory functions were upregulated. Taken together, parasites clearly possess an intrinsic ability to sense nutrient deprivation [11], but autophagy may have a context-dependent cytoprotective function.

Surprisingly upon drug administration artemisinin (ART)-resistant parasites initially experience induced cellular stress, which then diminishes to promote parasite regrowth [15] consistent with induced autophagy having a cytoprotective role in *Plasmodium*. Genome-wide analysis of ART-resistant field isolates revealed both mutations in and differential expression of several autophagy genes [11, 16, 17]. In addition, drug-resistant parasites also exhibit decreased expression or mutation of *Pf*Kelch13 (K13) and elevated levels of phosphatidylinositol 3-kinases (PI3K) and PI3P (significant inducers for autophagosome formation) [18–20]. Although the ubiquitin-proteasomal pathway is known to be enhanced in ART-resistant parasites [21, 22], activation of autophagy was not reported.

Herein, we addressed whether ART-resistant parasites induce autophagy as a pro-survival response and whether targeting this cytoprotective function could re-sensitize drug-resistant parasites. We demonstrated that resistant parasites exhibit a heightened number of *Pf*Atg8-positive punctae characteristic of autophagosomes upon mild starvation. Changes in the number of *Pf*Atg8-positive autophagosomes also correlated with parasite survival, growth, and any alteration in basal autophagy was not well tolerated by *Pf*K13 mutant parasites. Furthermore, inhibiting basal autophagy in resistant parasites led to growth impairment, thereby unveiling autophagy regulators, as a new therapeutic target to combat *Plasmodium* artemisinin resistance.

## Result

### Elevation of basal PfAtg8 expression in ART-resistant parasites

**Fig. 1:**
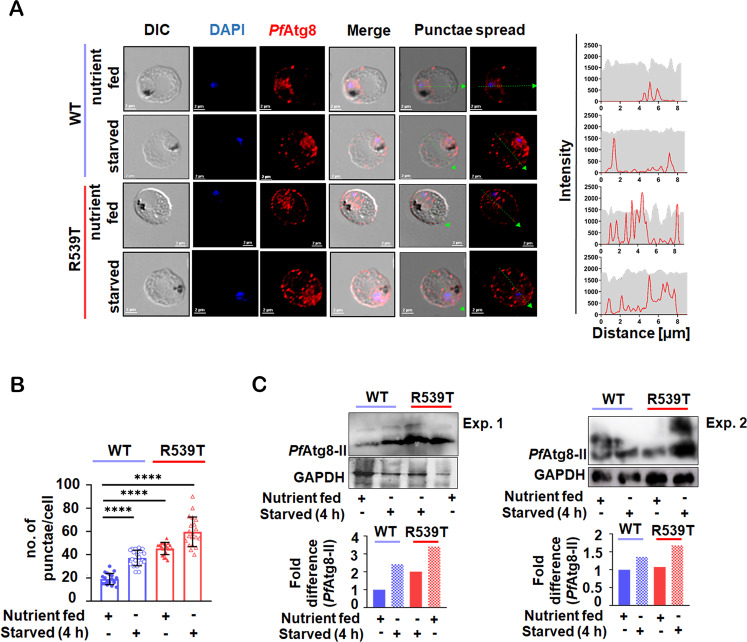
Induced basal autophagy in ART-resistant parasites. **A** In vitro ART sensitive (WT) and resistant (R539T) parasite-infected red blood cells (iRBC) were treated under nutrient-fed and nutrient-deprived (starved) medium for 4 h. iRBC were fixed post-treatment and immunolabelled with *Pf*Atg8 antibody (1:50) followed by anti-rabbit-Alexa 594 (1:300) (red). Nuclei were stained with DAPI (blue). The distribution of *Pf*Atg8 punctae in iRBC for each condition is represented as a line intensity profile. Scale bar: 2μm. **B** Quantitation of the number of *Pf*Atg8 punctae per iRBC (*n* = 20 cells were counted in each condition) from three independent experiments. **C** Synchronized trophozoite stage parasite lysates were prepared under nutrient-rich and starved conditions. *Pf*Atg8-II expression was analyzed by immunoblotting. GAPDH was used as a loading control. The expression of *Pf*Atg8-II was normalized based on the level of GAPDH per sample. Graphical representation indicates the fold difference in *Pf*Atg8-II expression compared to the normalized control. Uncropped blots are shown in Supplementary Fig. S2. Values represent mean ± SD, and ANOVA analysis with multiple comparisons was performed to calculate *p* values. *p value<0.05; **p value < 0.01; ***p value < 0.001; ****p value < 0.0001.

Reduced basal autophagy often results in impaired removal of toxic wastes [23], while upregulated basal autophagy is often associated with drug resistance and improved cellular survival [24, 25]. To elucidate the extent of autophagy at the trophozoite stage of parasite intraerythrocytic development we compared *Pf*Atg8 expression in wild-type (WT) and resistant (R539T) parasites under both nutrient-fed versus starved conditions. *Pf*Atg8 expression was monitored via immunofluorescence assay (IFA), where the average number of punctae per cell (*n* = 20) and pattern within infected erythrocytes were measured (Fig. 1A). *Pf*K13-R539T mutant parasites showed induced levels of basal autophagy when in a nutrient-fed state compared to the wild-type control (Fig. 1A). Remarkably, increase in the number of *Pf*Atg8 punctae per cell (*n* = 20) in *Pf*K13-R539T mutant parasites was more pronounced by ~2-fold and ~3-fold under basal and starvation conditions (Fig. 1B). While ~1-fold increase in the number of *Pf*Atg8 punctae per cell was observed in WT parasites under starvation conditions. Furthermore, the line intensity profile in Fig. 1A represents the spread of *Pf*Atg8-positive punctae across a single cell in both WT and R539T under complete and starvation conditions. *Pf*Atg8 decorated vesicles localized primarily in the parasite cytosol in WT parasites under steady-state conditions. By contrast, in *Pf*K13-R539T mutant parasites, *Pf*Atg8-positive vesicles predominantly localized proximal to the infected erythrocyte membrane with only a few in the parasite cytosol. Upon starvation of *Pf*K13-R539T mutant parasites, *Pf*Atg8-positive vesicles were greater in number and dispersed within parasite-infected erythrocytes (Fig. 1A). Additionally, the absence of any signal in the erythrocyte (RBC) lysate confirms the *Plasmodium* specific binding affinity of the anti-Atg8 antibody (Supplementary Fig. S4).

In parallel, we also confirmed autophagy induction by western blotting. Under nutrient-fed conditions, *Pf*K13-R539T mutant parasites showed increased *Pf*Atg8-II levels compared to WT parasites (Fig. 1C). Notably, in *Pf*K13-R539T mutant parasites growing under basal conditions the amount of Atg8-II was similar to WT parasites growing in a nutrient-limited environment. Figure 1C shows that starvation (4 h) of *Pf*K13-R539T mutant parasites causes a significant increase in amounts of Atg8-II compared to WT parasites based on three separate blots using three different cell extracts and scanned individually. Thus, under basal conditions, the *Pf*K13-R539T mutant background appears to trigger an autophagy-like mechanism in ART-resistant parasites.

### Induced basal autophagy correlates with survival in ART-resistant parasites

**Fig. 2:**
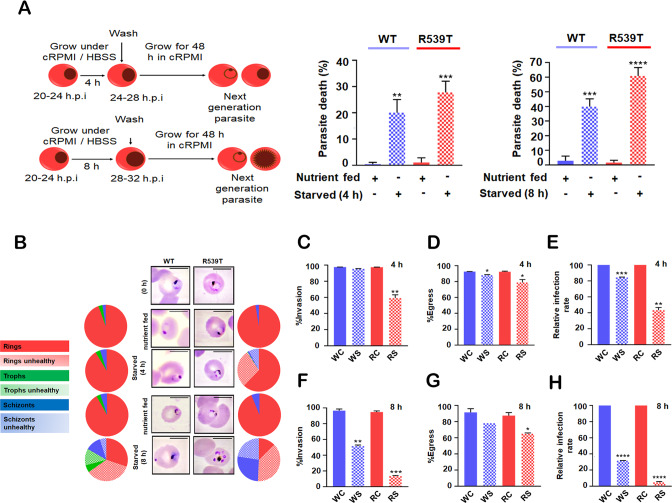
Stimulating autophagy above basal level abrogates ART-resistant parasite survival. **A** The schematic diagram for the experimental protocol. Synchronized early trophozoite stage iRBC, both WT and R539T, were incubated in nutrient-rich and starvation medium for 4 and 8 h, and parasitemia was determined after 48 h. Values represent asexual blood-stage (ABS) parasite death determined by flow cytometry after staining with EtBr (*n* = 3/condition, with a total of 100,000 events/samples/experiment). **B** Giemsa-stained smears showing parasite morphology under different treatment conditions. The pie chart depicts relative proportions of both healthy and unhealthy rings, trophozoites, and schizonts during different conditions. **C**, **F** The graph represents the percentage of next- generation rings invading fresh erythrocytes post 48 h for both WT and R539T parasites under nutrient-fed and starved conditions (4 and 8 h). **D**, **G** Graph representing the percentage of schizonts egressed post 4 and 8 h treatment under nutrient-fed and starved conditions for both WT and R539T parasites. **E**, **H** Graph represents the infectivity rate of erythrocytes by the parasites (both WT and R539T) post 48 h treatment under starving and nutrient-rich conditions. Nearly 4000 parasites were scored for analysis in an unbiased manner. Error bars show standard deviation. Unpaired student t-test was performed to calculate *p* values. *p value < 0.05; **p value < 0.01; ***p value < 0.001.

To further investigate the effects of nutrient limitation in drug-resistant parasites, we starved WT and ART-resistant parasites (early trophozoite stage) for a short time (4 h). Nutrient-fed parasites were used as the control for monitoring normal parasite growth. As previously reported [13], WT parasites at the trophozoite stage led to ~20% reduction in their parasite load, while ~30% suppression in parasitemia was observed in *Pf*K13-R539T mutant parasites (Fig. 2A). Microscopic analysis using Giemsa staining revealed attenuated parasite morphology with ~40% reduced invasion of next-generation rings in *Pf*K13-R539T mutant parasites upon starvation, while WT parasites appeared morphologically healthy (Fig. 2B, C). Concomitantly, the 40% reduction in invasion was made up by ~30% unhealthy rings and ~8% non-egressed shrunken/dead schizonts (Fig. 2D). By following schizont egress per invading ring we found a ~15% reduction in infection rate through the IDC for WT parasites. Interestingly, we found that *Pf*K13-R539T mutant parasites had ~50-55% reduction in infectivity upon 4 h starvation (Fig. 2E). This observation confirmed our immunoblot and immunofluorescence assays that ART-resistant parasites maintain an induced level of basal autophagy. The results also highlight the sensitivity of *Pf*K13-R539T mutant parasites towards changes in their basal level of autophagy. Since autophagy can provide both cytoprotective and cytodestructive functions, we further investigated the effect of prolonged starvation in both wild-type and *Pf*K13-R539T mutant parasites. Early trophozoite stage parasites were subjected to 8 h starvation since extended starvation has been shown to be fatal for parasites [9, 13].

Consistent with previous reports, prolonged starvation in WT parasites resulted in ablated growth, reducing total parasitemia by ~40%. However, when *P*fK13-R539T mutant parasites are starved for 8 h, there was significantly reduced parasitemia by ~55% (Fig. 2A). Further microscopic examination revealed that ~60% of the surviving mutant parasites presented a typical crisis morphology (Fig. 2B, C). We measured the percent of invading rings and percent of egressing schizonts upon 8 h starvation and observed ~50% reduction in next-generation rings with 23% blockage in schizont egress. Substantial defects in invasion (~85%) and egress blockade (~35%) suggest that prolonged starvation may be triggering excessive autophagy-mediated cell death (Fig. 2F, G). Since *Pf*K13-R539T mutant parasites already show a higher level of basal autophagy, abrogated parasite growth or a 6-fold decrease in infectivity upon a further induction of autophagy (Fig. 2H) are consistent with a dual role as a pro-survival and pro-death mechanism. Together these data indicate that ART-resistant parasites may utilize autophagy as a pro-survival strategy.

### Targeting *Pf*PI3K results in a diminution in autophagic flux that impedes autophagy

**Fig. 3:**
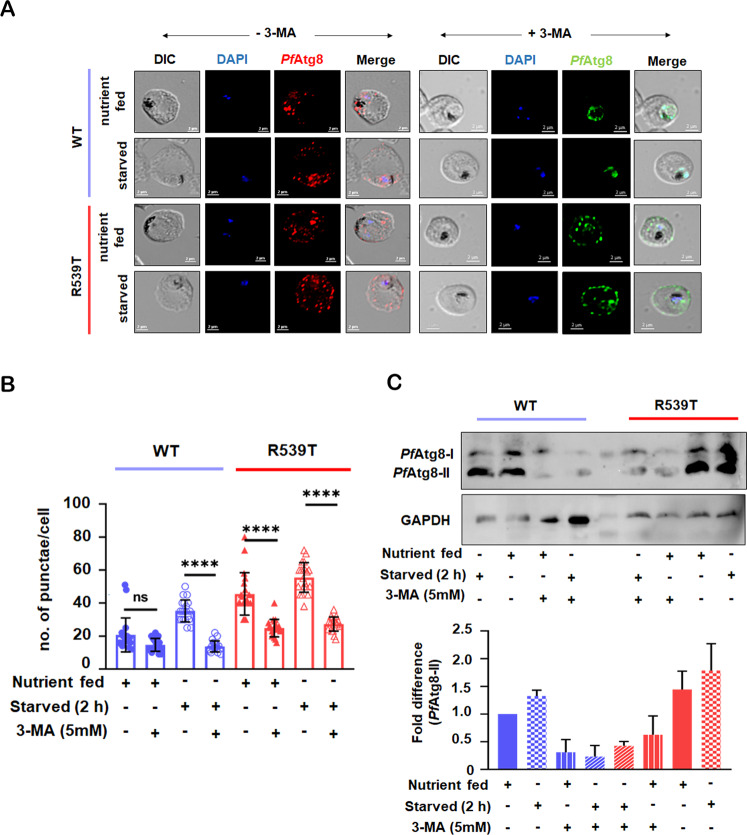
Targeting basal autophagy sensitizes ART-resistant parasites. **A** Synchronized WT and R539T iRBC were treated under nutrient-fed, starved medium, with and without 3-MA (5 mM) for 2 h. iRBC were fixed post-treatment and immunolabelled with *Pf*Atg8 antibody (1:50) followed by anti-rabbit-Alexa 594 (1:300) (red - without 3-MA) and anti-rabbit-Alexa-488 (1:500) (green - with 3-MA), respectively. Nuclei were stained with DAPI (blue). Scale bar: 2 μm. **B** Quantitation of the number of *Pf*Atg8 punctae per iRBC (for untreated iRBC and 3-MA treated iRBC, where n = 20 cells were counted in each condition). **C** Synchronized trophozoite stage parasite lysates were prepared under nutrient-rich, starved conditions in the presence and absence of 3-MA. *Pf*Atg8-II expression was determined by immunoblotting. GAPDH was used as a loading control. The expression of *Pf*Atg8 was normalized based on the level of GAPDH per sample. Graphical representation indicates the fold difference in *Pf*Atg8-II expression compared to the normalized control. Uncropped blot is shown in Supplementary Fig. S3. Values represent mean ± SD, and ANOVA analysis with multiple comparisons was performed to calculate *p* values. *p value<0.05; **p value < 0.01; ***p value < 0.001; ****p value < 0.0001.

When examining autophagy, measurements of flux either by blocking autophagosome formation or blocking the maturation and degradation of autophagosomes [26, 27] are more desirable. One standard approach is to block autophagosome formation using non-specific PI3K inhibitors such as 3-methyladenine (3-MA), LY294002, or wortmannin [26, 27]. To investigate whether inhibition of *Pf*PI3K (PF3D7_0515300) blocks parasite autophagic flux, we treated both WT and *Pf*K13-R539T mutant parasites with 3-MA under nutrient-fed versus nutrient-deprived conditions and followed the subcellular distribution of *Pf*Atg8. Microscopic quantification of *Pf*Atg8 punctae in WT and *Pf*K13-R539T mutant parasites showed that under basal conditions, 3-MA treatment led to a significant reduction in the dispersed localization of *Pf*Atg8 and a ~1-fold decrease in the average number of punctae per cell (*n* = 20 for 3-MA untreated and 3-MA treated parasites) in *Pf*K13-R539T mutant parasites (Fig. 3A, B). Interestingly, in *Pf*K13-R539T mutant parasites cultured under steady-state conditions, the expression of *Pf*Atg8 dropped to nearly that of WT parasites. To further confirm the modulation of autophagic flux, we performed western blot analysis under the same conditions. Figure 3C also shows that treatment with 3-MA significantly reduced *Pf*Atg8-II levels in *Pf*K13-R539T mutant parasites (based on two separate blots using two different cell extracts and scanned individually). Thus, both immunoblot and immunofluorescence sustain the notion that PI3K inhibition leads to an impediment of autophagy that alters autophagic flux in ART-resistant parasites similar to levels of WT parasites.

We also examined the effect of 3-MA on flux during starvation-induced autophagy. As shown in Fig. 3B, incubation of WT parasites with 3-MA inhibited the activity of *Pf*PI3K (as witnessed by a significant ~2.5-fold reduction in autophagosomes formed). Modulation of autophagy by starvation was again more pronounced in *Pf*K13-R539T mutant parasites, manifested by a significant decrease in *Pf*Atg8 punctae (*n* = 20 for 3-MA untreated and treated parasites). One can conclude that enhanced autophagic flux elicited by starvation was severely reduced by 3-MA treatment. Diminution of *Pf*Atg8 and autophagic flux was also measured by two separate blots using two different cell extracts and scanned individually to reveal a severe reduction in *Pf*Atg8-II levels in *Pf*K13-R539T mutant parasites (Fig. 3C). This strengthens the notion that *Pf*K13-R539T mutant parasites exhibit an induced level of basal autophagy that downregulates when the initiation of autophagy is pharmacologically blocked.

### Impairment of autophagy initiation leads to enhanced death of *Pf*K13-R539T mutant parasites

**Fig. 4:**
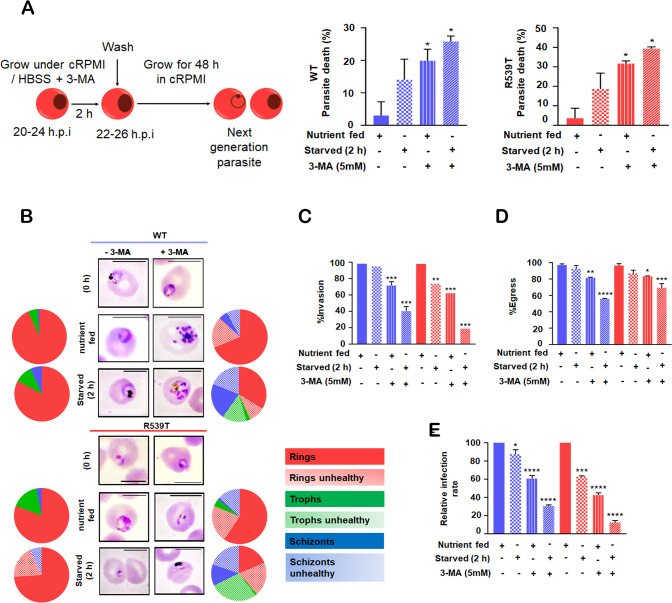
Inhibition of basal autophagy impedes autophagy-mediated pro-survival function in ART-resistant parasites. **A** The schematic diagram for the experimental protocol. Synchronized early trophozoite stage iRBC, both WT and R539T, were incubated in nutrient-fed or starved medium, with and without 3-MA (5 mM) for 2 h, and parasitemia was determined after 48 h. Values in the graph are asexual blood-stage (ABS) parasite death determined by flow cytometry after staining with EtBr (*n* = 2/ condition, with a total of 100,000 events/samples/experiment). **B** Giemsa-stained smears showing healthy and crisis parasite morphologies under different treatment conditions. The pie chart depicts relative proportions of both healthy and unhealthy rings, trophozoites, and schizonts during different conditions. **C** Graph represents the percentage of next-generation rings invading fresh erythrocytes post 48 h for both WT and R539T parasites cultured with or without starvation medium, in the presence or absence of 3-MA. **D** Graph representing the percentage of schizonts egressed post 48 h for both WT and R539T parasites cultured with or without starvation medium, in the presence or absence of 3-MA. **E** Graph represents the infectivity rate of erythrocytes by the parasites (both WT and R539T) post 48 h treatment under starving and nutrient-rich medium, in the presence or absence of 3-MA. The number of parasites scored for analysis, *n* = 4000, in an unbiased manner. Error bars show standard deviation, and ANOVA analysis with multiple comparisons was performed to calculate *p* values. *p value < 0.05; **p value < 0.01; ***p value < 0.001.

Early trophozoite stage WT and *Pf*K13-R539T mutant parasites were treated for 2 h with 3-MA under nutrient-rich conditions, and progression to the next intraerythrocytic cycle due to merozoite invasion of fresh erythrocytes was monitored by measuring parasitemia 48 h later (Fig. 4A). The parasitemia between nutrient-fed alone and nutrient-fed+3-MA was compared to evaluate the prerequisite of a basal level of autophagy for the survival of *Pf*K13-R539T mutant parasites. Nutrient-fed parasites with steady-state autophagy levels developed into merozoites that successfully invaded fresh erythrocytes and progressed to late rings and early trophozoites (Fig. 4B). On the contrary, WT and *Pf*K13-R539T mutant parasites treated with 3-MA, having a reduced basal level of autophagy, experienced ~15% and ~30% reduction in parasitemia (Fig. 4A). Microscopic analysis via Giemsa staining showed a pronounced effect of 3-MA on *Pf*K13-R539T mutant parasites (representative image shown in Fig. 4B). Following 3-MA treatment under nutrient-rich conditions, the WT parasite population had shrunken rings with ~30% reduced invasion and ~20% arrested schizonts with 40% reduced infectivity rate (Fig. 4C–E). By contrast, *Pf*K13-R539T mutant parasites had shrunken/compromised rings with ~40% reduced invading rings, ~20% schizonts with crisis morphology and 58% reduced infectivity rate (Fig. 4C–E). Thus, explaining the dependency of *Pf*K13-R539T mutant parasites on the cytoprotective function of autophagy exhibited by induced basal autophagy level.

Interestingly, upon starvation triggered autophagy the effect of inhibiting PI3K with 3-MA was more pronounced in *Pf*K13-R539T mutant parasites, indicating they are more sensitive to loss of basal autophagy. Starved WT parasites exhibited a ~25% reduction in growth, while R539T parasites suffered a ~40% drop in parasitemia (Fig. 4A). Morphological analysis revealed that ~ 40% of the rings progressed to the next cycle in WT parasites, and the rest appeared as arrested schizonts and unhealthy parasites, associated with 30% infectivity rate through the IDC (Fig. 4C–E). Importantly, quantitation of starved *Pf*K13-R539T mutant parasites treated with 3-MA revealed 80% reduction in the ring population of the next intraerythrocytic cycle (Fig. 4C) and compromised trophozoites, schizonts, and shrunken rings with zero invasion fitness (Fig. 4B, D). Severely compromised infection rate (~12%) in *Pf*K13-R539T mutant parasites depicts lethal defects in egress/invasion of these parasites upon abrogating their basal autophagy (Fig. 4E). Thus, targeting autophagy initiation, especially the primary mediator *Pf*PI3K, facilitates enhanced killing of *Pf*K13-R539T mutant ART-resistant parasites. Furthermore, our data strengthen the plausibility that upregulation of *Pf*PI3K and *Pf*PI3P in *Pf*K13-R539T mutant parasites facilitates induced basal autophagy levels, conferring on mutants a pro-survival function, making them more stress-tolerant and able to survive transient translational repression and dormancy.

### Abrogating autolysosome formation leads to incomplete autophagy and increased *Pf*Atg8 expression in *Pf*K13-R539T mutant parasites

**Fig. 5:**
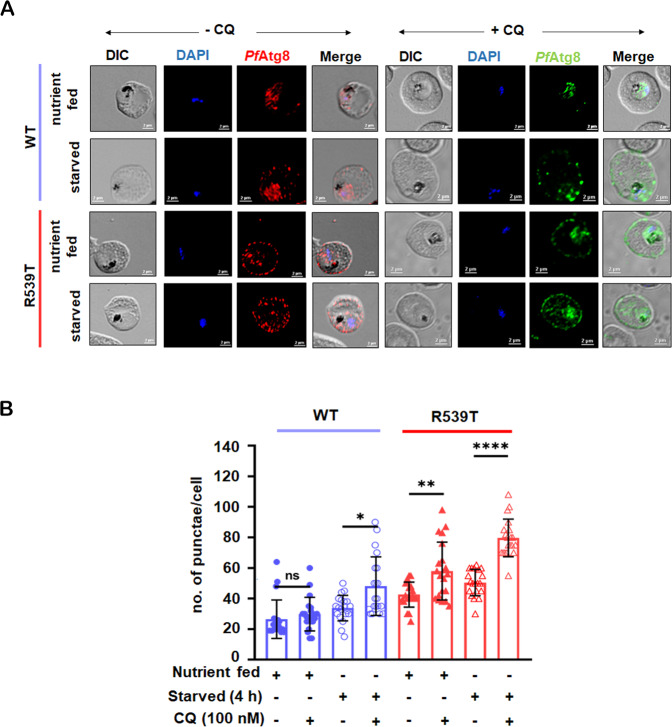
Enriched autophagic flux in ART-resistant parasites. **A** Synchronized WT and R539T iRBC were cultured in a nutrient-rich, starved medium, with and without CQ (100 nM), for 4 h. iRBC were fixed post-treatment and immunolabelled with *Pf*Atg8 antibody (1:50) followed by anti-rabbit-Alexa 594 (1:300) (red - without CQ) and anti-rabbit-Alexa-488 (1:500) (green - with CQ), respectively. Nuclei were stained with DAPI (blue). Scale bar: 2μm. **B** Quantitation of the number of *Pf*Atg8 punctae per iRBC in the presence and absence of CQ. The graph displays modulation in autophagic flux in both types of iRBC (for CQ untreated iRBC and CQ treated iRBC, *n* = 20 cells were counted for each condition). Values represent mean ± SD, and ANOVA analysis with multiple comparisons was performed to calculate *p* values. *p value < 0.05; **p value < 0.01; ***p value < 0.001; ****p value < 0.0001.

To negate the off-target effect of PI3K inhibitors on both class I and class III PI3K it’s essential to measure autophagic flux using lysosomotropic agents such as chloroquine (CQ) bafilomycin, etc [27]. Treatment of both WT and *Pf*K13-R539T mutant parasites at the trophozoite stage with CQ under nutrient-rich conditions resulted in condensed cytosolic expression of *Pf*Atg8-II centred around hemoglobin (black-pigmented spot) (Fig. 5A). However, the number and distribution of *Pf*Atg8 punctae representing the number of autophagosomes increased significantly in *Pf*K13-R539T mutant parasites. The increase in autophagosomes in the presence of CQ is consistent with autophagic flux. Starvation of WT parasites led to a redistribution of *Pf*Atg8-labeled punctae indicative of increased autophagic flux that slightly increased in the presence of CQ. However, *Pf*K13-R539T mutant parasites exhibited more *Pf*Atg8-positive autophagosomes in the presence of CQ, clustering mainly near the food vacuole with an additional number around the erythrocyte plasma membrane and few accumulated within the infected erythrocyte (Fig. 5B). Altogether, the data support a greater dependency of *Pf*K13-R539T mutant parasites on autophagy; both as a pro-survival and cellular degradation mechanism.

### Potent and selective class III PI3K inhibitors such as SAR405 act as a potential druggable lead against ART-resistant parasites

To assess whether a potent, selective PI3KC3 inhibitor can be exploited as a novel drug against ART-resistant parasites, we tested the human Vps34 (hPI3KC3)-specific inhibitor, SAR405 as a pharmacological tool [28]. An overlay of human Vps34 and *Pf*PI3K amino acid sequences revealed similar geometry for the kinase domain and ATP-binding site (Supplementary Fig. S1A). In silico docking studies with SAR405 revealed three *Pf*PI3K amino acid residues, LEU1841, LEU1886, and LEU2011, with hydrophobic interactions. Likewise, LYS2076 and LEU1881 formed a hydrogen bond with 2.03 Å and 2.21 Å H-bond distance (Supplementary Fig. S1B).SAR405 docked into the ATP-binding pocket with a binding energy of −6.24 kcal/mol.

**Fig. 6:**
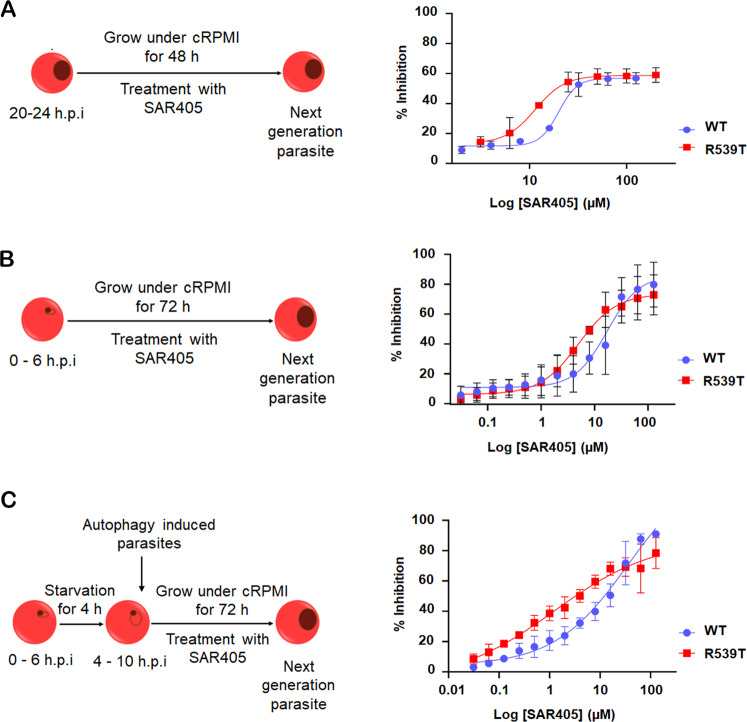
Targeting *Pf*PI3K inhibits the cytoprotective role of autophagy and re-sensitizes ART-resistant parasites. **A**–**C** The schematic diagram of the experimental protocol. **A**, **B** In vitro grown ART sensitive (WT) and resistant (R539T) iRBC were incubated with increasing concentrations (0–128 μM) of selective PI3KC3 inhibitor, SAR405, in nutrient-rich media for 48 or 72 h. Growth inhibition assays were performed to determine the inhibitory concentration (IC_50_ values) in both early trophozoite (**A**) and early ring-infected RBC (**B**). Analysis of viable parasites was done by measuring parasitemia via flow cytometry. **C** Dose-dependent effect of SAR405 on iRBC under starvation conditions. iRBC were cultured for 4 h in a starvation medium, followed by treatment with increasing concentrations of SAR405 (0–128 μM) in a nutrient-rich medium. IC_50_ values were determined by measuring parasitemia using flow cytometry (total of 100,000 events/samples/experiment). Data are representative of two independent experiments, and values represent mean ± SD.

Furthermore, treatment of infected red blood cells dampened *Pf*PI3K activity suppressed induced autophagy and altered *Pf*K13-R539T mutant parasite growth in vitro (Fig. 6A). The dose-response curve for SAR405 activity against early ring stage WT and *Pf*K13-R539T mutant parasites yielded IC_50_ values of 17.1 ± 1.71 µM and 4.9 ± 2.68 µM, respectively (Fig. 6B). The stage-specific inhibitory effect of SAR405 was more pronounced for *Pf*K13-R539T mutant parasites, conferring a 3.5-fold shift in the IC_50_ compared to WT parasites (Supplementary Table 1).

We investigated if the above sensitivity was due to impaired autophagic flux, resulting in a reduced level of basal autophagy in *Pf*K13-R539T parasites. Young ring-stage parasites were starved for 4 h followed by treatment with SAR405 and growth was monitored post-48 h. Although starvation would provoke normal autophagic flux, treatment with SAR405 would inhibit *Pf*PI3K altering the autophagic flux in *Pf*K13 mutant parasites. Growth inhibition assays showed the IC_50_ dropped from 4.9 ± 2.68 µM to 1.2 ± 0.93 µM in *Pf*K13-R539T mutant parasites (Fig. 6C). As shown in Supplementary Table 1, an increase in the IC_50_ value of SAR405 by ~0.5-fold was noted for WT parasites. Notably, stage-specific sensitivity was not evident in both the trophozoite and ring stages of WT parasites. The ~4-fold shift in IC_50_ for starved *Pf*K13-R539T mutant parasites is consistent with an abatement of autophagic activity due to SAR405 inhibiting *Pf*PI3K. The heightened sensitivity of *Pf*K13-R539T mutant parasites towards suppression of basal autophagy was mirrored by reduced numbers of *Pf*Atg8 punctae during treatment with SAR405 (data not shown), targeting both autophagy initiation and autolysosome-mediated degradation.

## Discussion

Here, we studied the contribution of autophagy to artemisinin-resistant malaria parasites. We undertook a careful analysis of autophagy in K13 ART-resistant and wild-type parasites and chose an ART-resistant mutant parasite *Pf*K13-R539T since this particular mutant survives best ART-drug pressure in vitro [17]. Different parameters (*Pf*Atg8 expression, autophagic flux, and parasite growth) and pharmacological tools (autophagy inhibitors) were used, and the ensemble of results underscores the propensity of ART-resistant parasites to enhance autophagy under basal conditions, demonstrating a pro-survival mechanism similar to cancer cells [4, 24, 25].

During asexual WT parasite growth, *Pf*K13 is located at both ends of the cytostome, where it assists in engulfing host cell content that is trafficked to and digested in the food vacuole to supplement the nutritional requirements for parasite growth [15]. Importantly, ART-resistant parasites have a ~50% reduction in *Pf*K13 protein abundance that results in reduced hemoglobin endocytosis, limited nutrient supply, and increased dependency on an exogenous source of nutrients [29, 30]. This is consistent with a greater dependency of *Pf*K13 mutant ART-resistant parasites on autophagy than WT parasites. Similar to previous reports [9, 13], we observed that parasites respond to nutrient starvation by (i) upregulating their basal autophagy, (ii) stimulating autophagic flux that (iii) permits parasite growth with little impact on parasitemia during mild starvation (Figs. 1 and 2). Prolonged starvation, however, resulted in reduced parasitemia and stalled parasite growth (Fig. 2) [9, 12]. Surprisingly, the effect of nutrient deprivation was more lethal for ART-resistant parasites. As depicted in Fig. 1, in *Pf*K13-R539T mutant parasites *Pf*Atg8-positive autophagosomes are dispersed in the parasite cytoplasm and decorate the erythrocyte membrane with an enhanced autophagic flux. One explanation could be to regulate the exportome [18], thereby maintaining the cytoadherence property of mutant parasites. Another could be the uptake of exogenous nutrients to mitigate proteopathic damage via an autophagy-like mechanism [18, 31].

We observed that blocking upstream steps of ALP with 3-MA had a dire effect on the growth of *Pf*K13-R539T parasites compared to WT parasites (Fig. 4). The significant curtailment of autophagic vesicles labelled with *Pf*Atg8, their altered distribution, and turnover of *Pf*Atg8 lipidation in the presence of 3-MA in *Pf*K13-R539T mutant parasites (Fig. 3) strongly argues for the existence of an autophagy-like mechanism in ART-resistant parasites. Furthermore, the impact of abrogating upstream initiation of autophagy was more severe for resistant parasites (Fig. 4) and resulted in ablating their resistance to artemisinin by dampening the activity of *Pf*PI3K. Additionally, increased accumulation of autophagosomes in *Pf*K13-R539T mutant parasites under basal conditions in the presence of lysosomotropic agent chloroquine (CQ) further indicates abrogation of lysosome mediated degradation (Fig. 5). CQ treatment combined with nutrient deprivation had a more telling effect on resistant parasites, thereby representing increased autophagic flux than a block in fusion or ALP mediated degradation. Hence, increased activity of PI3K and thus PI3P, translational repression as an adaptive response, dependency on an exogenous source of nutrients due to lack of cytostome activity/hemoglobin uptake, and the imperative need to restore cellular homeostasis, combine to suggest autophagy as a principal backup mechanism in artemisinin-resistant parasites.

The single parasite phosphatidylinositol 3-kinase gene (PF3D7_0515300) is a homolog of mammalian and yeast PI3KC3/Vps34 and resembles them in terms of its activity being involved in endocytosis, nutrient uptake, and parasite growth [32, 33]. Therefore, we propose that augmented *Pf*PI3K activity induces autophagy in resistant parasites to compensate for damage incurred under stress conditions experienced during their development or drug treatment. We utilized a chemotherapeutic approach to dampen *Pf*PI3K-dependent autophagy-mediated pro-survival strategy in *Pf*K13-R539T parasites. The specific PI3KC3 inhibitor SAR405 proved more effective against *Pf*K13-R539T mutant than WT parasites (Fig. 6) and re-sensitized artemisinin-resistant early ring-stage parasites (Fig. 6B and C). On the contrary, WT parasites with lower PfPI3K expression and PI3P levels remain indifferent to the stage-specific effect of SAR405. Recently, tailoring *Pf*PI3K activity has also been shown to modulate a signaling cascade (presumably autophagy-like) in CQ-resistant parasites [33]. Hence, autophagy may act as a peripheral pro-survival pathway favouring the persistence of drug-resistant malaria parasites. The intimate connection between *Pf*PI3K and autophagy in *Pf*K13-R539T mutant parasites illustrated in this study highlights future avenues for autophagy and/or PI3-Kinase inhibitors to combat drug resistance in *Plasmodium*. Finally, our study highlights the existence of a semi-starved metabolic state and a precise cytoprotective mechanism behind the pro-survival strategy adopted by artemisinin-resistant parasites to better tolerate stress.

## Materials and methods

### *P. falciparum* culture

*P. falciparum* 3D7 parasite lines bearing either wild-type artemisinin-sensitive marker *Pf*Kelch13^WT^ (WT) or artemisinin-resistant mutation *Pf*Kelch13^R539T^ (R539T) were generated as described in Mbengue et al. [34]. Parasites were provided by Souvik Bhattacharjee (Jawaharlal Nehru University, Delhi, India). As described previously [35] parasites were maintained in RPMI 1640 (Gibco) supplemented with 27.2 μg/mL of hypoxanthine (Sigma), 5 μg/L Gentamycin (Sigma), and 0.5% Albumax I (Gibco), using O+ RBCs (2% hematocrit) in a mixed gas environment (5% O2, 5% CO2 and 90% N2). Parasites were tightly synchronized using 5% sorbitol (Sigma) and 65% Percoll (GE Healthcare). For culturing, O+ RBCs were obtained from the Rotary blood bank, Delhi.

### Starvation of infected red blood cells

For starvation experiments, infected red blood cells harbouring highly synchronized WT and R539T schizonts stages were purified using Percoll density gradients followed by tight synchronization using sorbitol. The tightly synchronized parasite-infected red blood cells were allowed to grow for one cycle at low parasitemia to keep them stress-free. In the next cycle, at the early trophozoite stage (20–24 h), synchronized parasite-infected red blood cells were cultured either in nutrient-rich (complete RPMI 1640 medium) or nutrient-deprived/starvation medium [Hank’s balanced salt solution (HBSS)-Himedia] for 4 or 8 h duration at 37 °C under the same gaseous condition. Parasite-infected red blood cells undergoing mild nutrient deprivation were starved for 4 h, while prolonged starvation was for 8 h.

### Parasite-infected red blood cell in vitro growth assay

To investigate the effect of starvation, tightly synchronized early stage trophozoite infected red blood cells (20–24 hpi) were supplemented with complete (nutrient-fed) and starved medium for 4 h or 8 h at 37 °C. Post-specific treatments, infected red blood cells were washed and cultured in a complete medium for one cycle. Parasites were stained with EtBr, and 100,000 events/samples were analyzed on a CytoFLEX S flow cytometer instrument (Beckman Coulter, USA). The data was further analyzed on FlowJo (TreeStar). Red blood cells were determined based on their size by gating on the forward light scatter (FSC)/side scatter (SSC) plot and the infected erythrocytes were simultaneously gated on the EtBr channel (PE Texas red/FL3). Uninfected erythrocytes did not contain any positive cells for dye, hence used as a negative control. WT and R539T parasite-infected red blood cells cultured in a complete medium served as a positive control. For morphological analysis, ~4000 parasite-infected red blood cells under both conditions were monitored and scored from Giemsa-stained blood smears. The smears of each sample in all individual experiments were blinded before scoring the parasites to avoid observer bias.

### Treatment with autophagy inhibitors (3-MA, CQ)

Modulation in the level of autophagy both upstream and downstream was determined by treating the parasite-infected red blood cells with different inhibitors. Briefly, highly synchronized infected red blood cells at their early trophozoite stage (20–24 h) were exposed to the following inhibitors: 3-methyladenine (3-MA, 5 mM), chloroquine (CQ, 100 nM). Parasite-infected red blood cells were incubated with 3-MA (Sigma) for 2 h under both control (control, control+3-MA) and starvation (starved, starved+3-MA) conditions and subsequently processed for immunofluorescence assay (IFA), western blotting, or growth assay. Post-treatment parasite-infected red blood cells were washed and cultured in a complete medium for one cycle for growth assays. Parasites were stained with EtBr, and 100,000 events/samples were analyzed on a CytoFLEX S flow cytometer instrument (Beckman Coulter, USA). The data was further analyzed on FlowJo (TreeStar). RBCs were determined based on their size by gating on the forward light scatter (FSC)/side scatter (SSC) plot, and infected erythrocytes were simultaneously gated on the EtBr channel (PE Texas red/FL3). Uninfected erythrocytes did not contain any cells positive for dye and were hence used as a negative control. WT and R539T parasite-infected red blood cells cultured in a complete medium served as a positive control. For morphological analysis, parasite-infected red blood cells under both conditions were monitored and scored from Giemsa-stained blood smears.

For studies with CQ (Sigma) treatment, parasite-infected red blood cells were treated for 4 h, under both control (control, control + CQ) and starvation (starved, starved + CQ) conditions and subsequently processed for immunofluorescence assay (IFA).

The percentage of parasite death was calculated as follows –

$${{{\mathrm{\% }}}}\;{{{\mathrm{parasite}}}}\;{{{\mathrm{death}}}} = 100 - \left( {\frac{{{{{\mathrm{parasitemia}}}}\;{{{\mathrm{of}}}}\;{{{\mathrm{test}}}}}}{{{{{\mathrm{parasitemia}}}}\;{{{\mathrm{of}}}}\;{{{\mathrm{control}}}}}} \ast 100} \right)$$

The relative infection rate was calculated as follows –

$${{{\mathrm{Invasion/egress}}}} = \frac{{{{{\mathrm{\% }}}}\;{{{\mathrm{rings}}}}\;{{{\mathrm{invasion}}}}\;{{{\mathrm{in}}}}\;{{{\mathrm{control}}}}\;{{{\mathrm{or}}}}\;{{{\mathrm{treated}}}}}}{{{{{\mathrm{\% }}}}\;{{{\mathrm{schizont}}}}\;{{{\mathrm{egressin}}}}\;{{{\mathrm{control}}}}\;{{{\mathrm{or}}}}\;{{{\mathrm{treated}}}}}}$$

The ratio of invasion/egress for parasites (both WT and R539T) grown in nutrient-fed medium was considered as positive control with 100% infection rate. The relative infection rate was calculated for parasites grown in starvation, starvation ±3-MA, and nutrient-fed ±3-MA with respect to their positive control.

### Immunofluorescence assay (IFA)

Synchronous *P. falciparum-* (WT and R539T)-infected red blood cells were cultured in a complete and starved medium for 4 h at 37 °C. Mid-trophozoite-infected red blood cells were fixed using a fixative solution (4% paraformaldehyde and 0.0075% glutaraldehyde in PBS) for 30 min at room temperature. Parasite-infected red blood cells were washed twice with PBS and permeabilized using 0.1% Triton X-100 at room temperature for 3 min. Further parasite-infected red blood cells were washed and blocked with 3% bovine serum albumin (BSA) for 1 h at room temperature. The parasite-infected red blood cells were incubated with a primary rabbit antibody raised to *Pf*Atg8 [9] (1:50) in 3% BSA for 1 h at room temperature followed by Alexa Fluor 594 anti-rabbit secondary antibody (Molecular Probes) at 1:300 dilution or Alexa Fluor 488-anti-rabbit secondary antibody (Molecular Probes) at 1:500 dilution for 1 h at room temperature. Parasite-infected red blood cells were mounted over a glass slide with ProLong Gold DAPI antifade (Molecular Probes). Confocal images were acquired using Nikon A1R MP + multiphoton confocal microscope using a 100× oil immersion objective. Serial Z sections of each image with similar acquisition parameters and step size of 0.2 µm were gathered, and the best representations are illustrated in the figures. Z-stack images were processed and deconvoluted for illustration via NIS-Elements software. Maximum intensity projection (MIP) of each parasite-infected red blood cell was used for scoring the number of punctae/cells, respectively. Approximately *n* = 20 parasites were counted each for control parasites (WT, R539T)-infected red blood cells under nutrient-fed and starved conditions, and for 3-MA or CQ treated parasites from three independent experiments.

### Atg8 western blotting

For immunoblotting, pellets of synchronized trophozoite stage parasites grown in a complete and starved medium in the presence and absence of 3-MA (5 mM) were collected. Parasites were separated from erythrocytes by saponin lysis (0.15% saponin in PBS) followed by washes with cold PBS. The parasites were suspended in an SDS-PAGE sample buffer for 30 min at room temperature and sonicated with three pulses of 15 s each at 20% amplitude. Lysates were centrifuged at 16,000 × *g* for 5 min, and the supernatant was collected. Protein in each sample was estimated using BCA protein assay. Around 30 µg protein samples were resolved on a 15% SDS-PAGE gel and transferred onto the PVDF membrane (Millipore). The membranes were blocked with blocking buffer (5% skimmed milk in PBS-tween20) for 2 h at room temperature followed by overnight incubation at 4 °C with primary antibody rabbit anti-*Pf*Atg8 (1:100) and mouse anti-GAPDH (1:1000) (Invitrogen, Carlsbad, CA, USA, 1:500) in blocking buffer. The blots were washed and probed with HRP-conjugated anti-rabbit and anti-mouse secondary antibody at dilution (1:2000 and 1:10,000) for 1 h at room temperature. The signals were developed with a western ECL kit (Bio-Rad) using Image-Quant LAS 500 (GE Healthcare).

For obtaining ghost erythrocyte lysate, uninfected erythrocytes were lysed by freeze-thawing in cold phosphate buffer (pH 8.0) and vortexed to isolate erythrocyte membrane. Samples were centrifuged at 20,000 × *g* for 20 min at 4 °C and erythrocyte membrane pellets were isolated. Followed by the pellets were washed with phosphate buffer to obtain ghost erythrocytes. All the western blots were repeated twice with individual sample preparation.

### In silico analysis

The protein structure of *P. falciparum* class III phosphatidylinositol 3-kinase (*Pf*PI3K) (PF3D7_0515300) was obtained from PlasmoDB. Phyre2 web server was used for homology 3D protein modeling of *Pf*PI3KC3 [36, 37]. Swiss PDB viewer and ChemBio Draw ultra 3D software were used to optimize protein and ligands structure [38]. Molecular docking of molecule SAR405 with *Pf*PI3K was performed using Autodock version 4.2 and Cygwin terminal version 3.1 software [39]. Residues present around the binding pocket were chosen to prepare a grid (spacing 0.375; npts 80 80 80; centre X-86.791 Y-79.021 Z-16.852) to perform ligand binding. Discovery Studio version 19.1.0 and Pymol version 2.3.2 software were utilized to analyze and visualize the docking results [40, 41].

### SAR405 treatment of parasite-infected red blood cells

Infected red blood cells harbouring early trophozoite stage (20–24 h) and early rings (0-6 h) stages of both the strains (WT and R539T) with starting parasitemia adjusted to 1% and 2% hematocrit were incubated with varying concentrations of SAR405 (Calbiochem) (0–200 μM) at 37 °C in a mixed gas environment for one cycle (48 h). Early ring-infected red blood cells treated with SAR405 were incubated for 72 h. Untreated infected red blood cells of both strains served as control. Following incubation, infected red blood cells were stained with EtBr, and 100,000 events/samples were analyzed on a CytoFLEX S flow cytometer instrument (Beckman Coulter, USA). The data was further analyzed on FlowJo (TreeStar). RBCs were determined based on their size by gating on the forward light scatter (FSC)/side scatter (SSC) plot and the infected erythrocytes were simultaneously gated on the EtBr channel (PE Texas red/FL3). Uninfected erythrocytes used for parasite culturing did not contain any positive cells for the dye, hence used as a negative control. In vitro, 50% inhibitory concentrations (IC_50_) were determined by nonlinear regression analysis using GraphPad (Prism).

Further, to evaluate the ability of SAR405 to sensitize the K13 mutant-infected red blood cells, early (0-6 h) ring stage-infected red blood cells were initially subjected to autophagy-inducing environment (starvation). Autophagy was induced by 4 h starvation of both WT and R539T parasite-infected red blood cells, while infected red blood cells supplemented with complete medium served as control. After their treatment, all the parasite-infected red blood cells were washed and replenished with complete media, parasitemia adjusted to 1% with 2% hematocrit, and then added to the plate having varying concentrations of SAR405 (0–128 μM). Post 72 h, parasite-infected red blood cells were stained with EtBr, and parasitemia was determined as described above via flow cytometry.

Kindly note SAR405 was only used as a pharmacological tool for the proof of concept. It is not recommended as a drug candidate but used to illustrate a new target against drug-resistant parasites for novel drug discovery.

### Statistical analysis

All multiple comparison experiments were analyzed by ANOVA using GraphPad Prism. Unpaired student’s *t*-test for unequal variances was performed to evaluate significant differences between control (nutrient-rich) and starved samples. *p value < 0.05; **p value < 0.01; ***p value < 0.001; ****p value < 0.0001 were considered significant. Results represent the mean ± SD of a minimum of two/three independent experiments unless otherwise stated. All IC_50_ values presented are reported with a 95% confidence interval.

## Supplementary information

Supplementary Table 1

Supplementary Figure 1

Supplementary Figure 2

Supplementary Figure 3

Supplementary Figure 4

## Data Availability

All data generated or analyzed during this study are included in this published article and its supplementary information files.
