# Supplementary material for: Cytoprotective autophagy as a pro-survival strategy in ART-resistant malaria parasites
Source: Cell Death Discov. 2023 May 13;9:160. doi: 10.1038/s41420-023-01401-5 (PMC10182036; doi:10.1038/s41420-023-01401-5)
Supplement: Supplementary file 1 — Supplementary Table 1 [file 41420_2023_1401_MOESM1_ESM.pdf]

Stage	SAR405 IC50 (µM)	
	WT	R539T
Trophozoite	19.90 ± 0.5	11.47 ± 0.43
Ring	17.11 ± 1.7	4.946 ± 2.68
Ring + Starvation	26.39 ± 1.5	1.237 ± 0.93
