# Supplementary figures and images for: Cytoprotective autophagy as a pro-survival strategy in ART-resistant malaria parasites
Source: Cell Death Discov. 2023 May 13;9:160. doi: 10.1038/s41420-023-01401-5 (PMC10182036; doi:10.1038/s41420-023-01401-5)

A

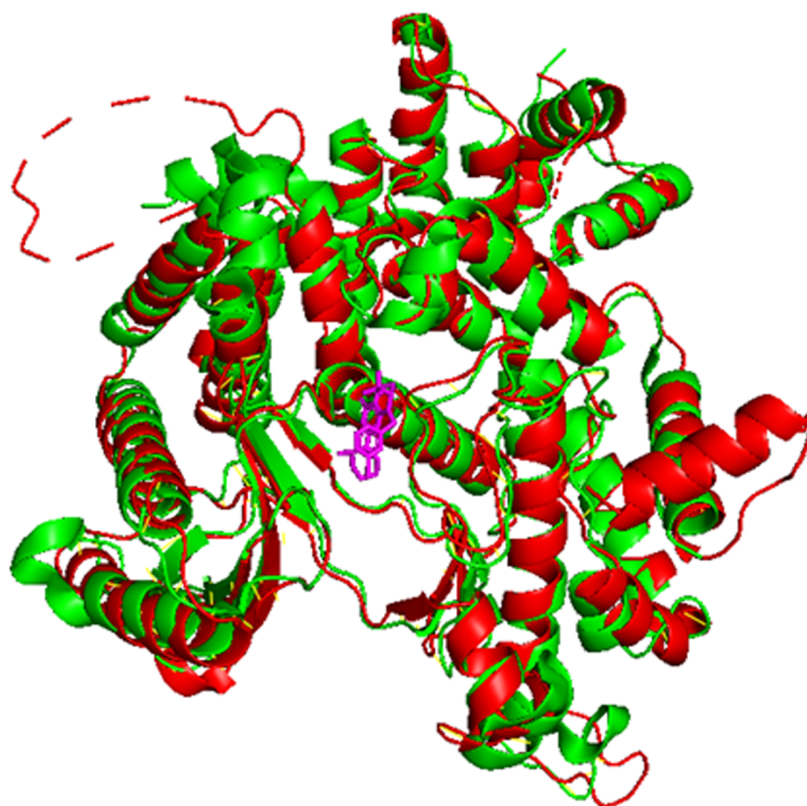

B

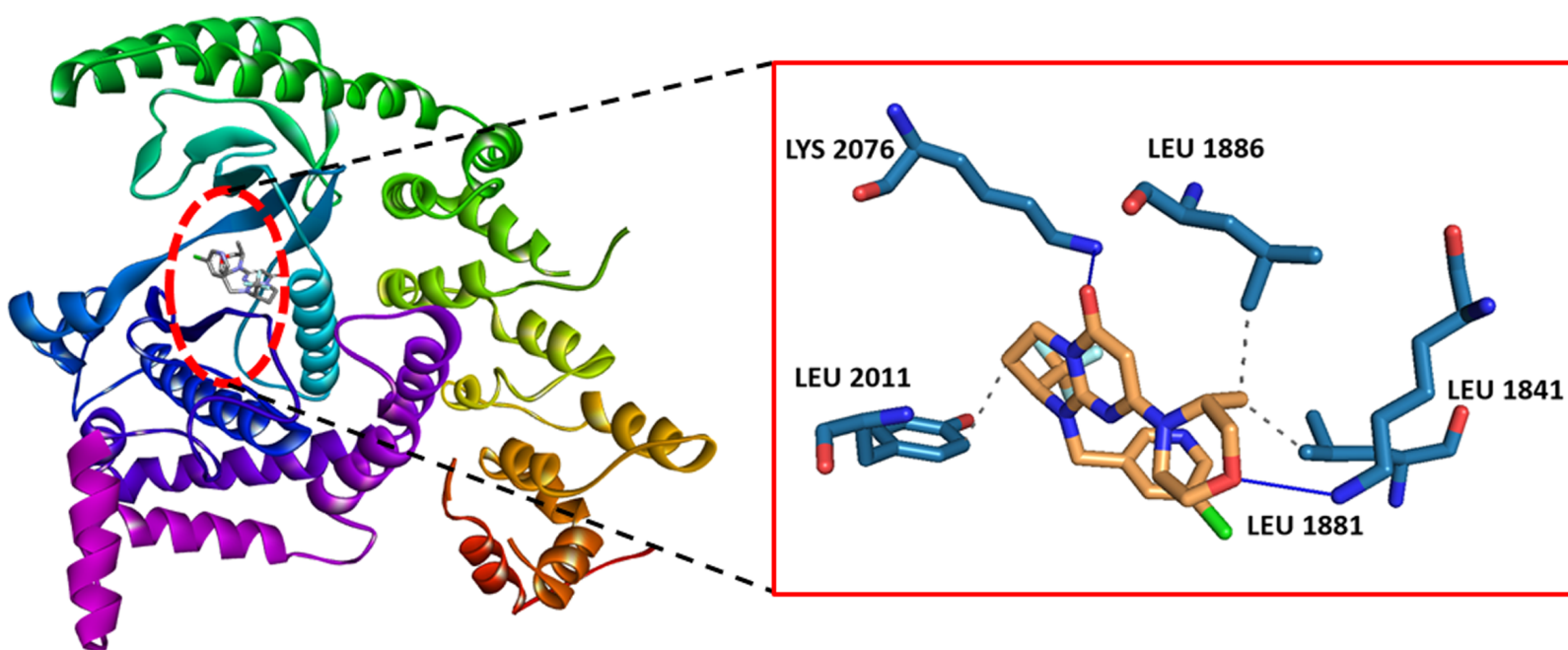

Supplementary figure 1. In silico analysis of SAR405 with PfPI3K

Supplement: Supplementary file 2 — Supplementary Figure 1 [file 41420_2023_1401_MOESM2_ESM.pdf]

**A**

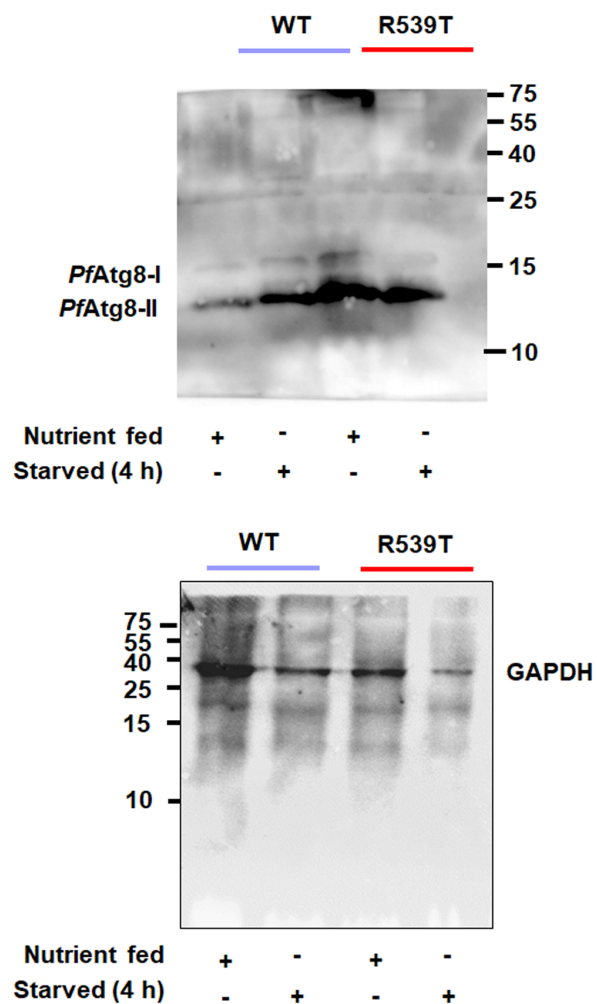

**B**

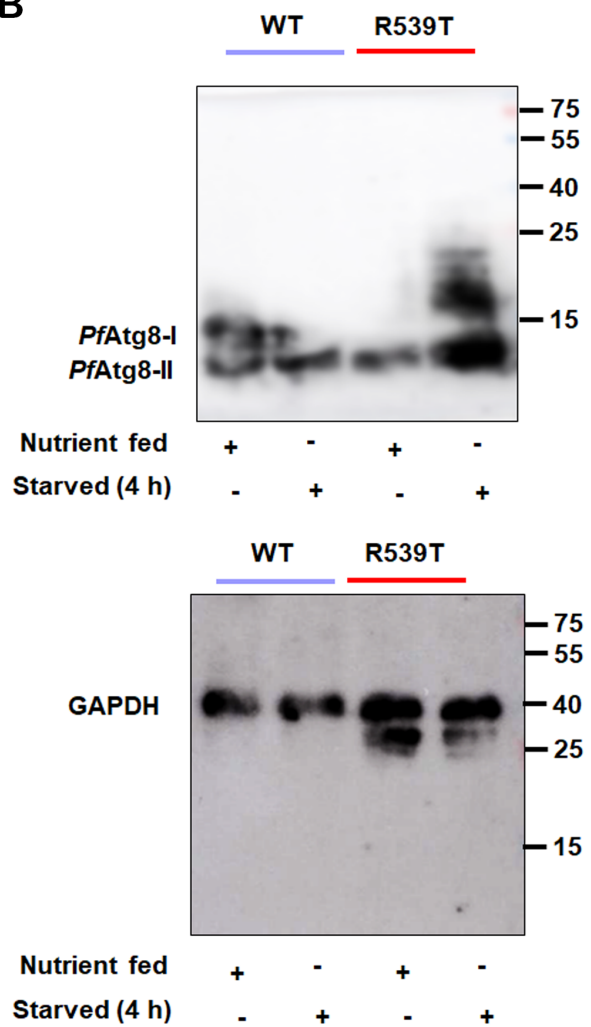

Supplement: Supplementary file 3 — Supplementary Figure 2 [file 41420_2023_1401_MOESM3_ESM.pdf]

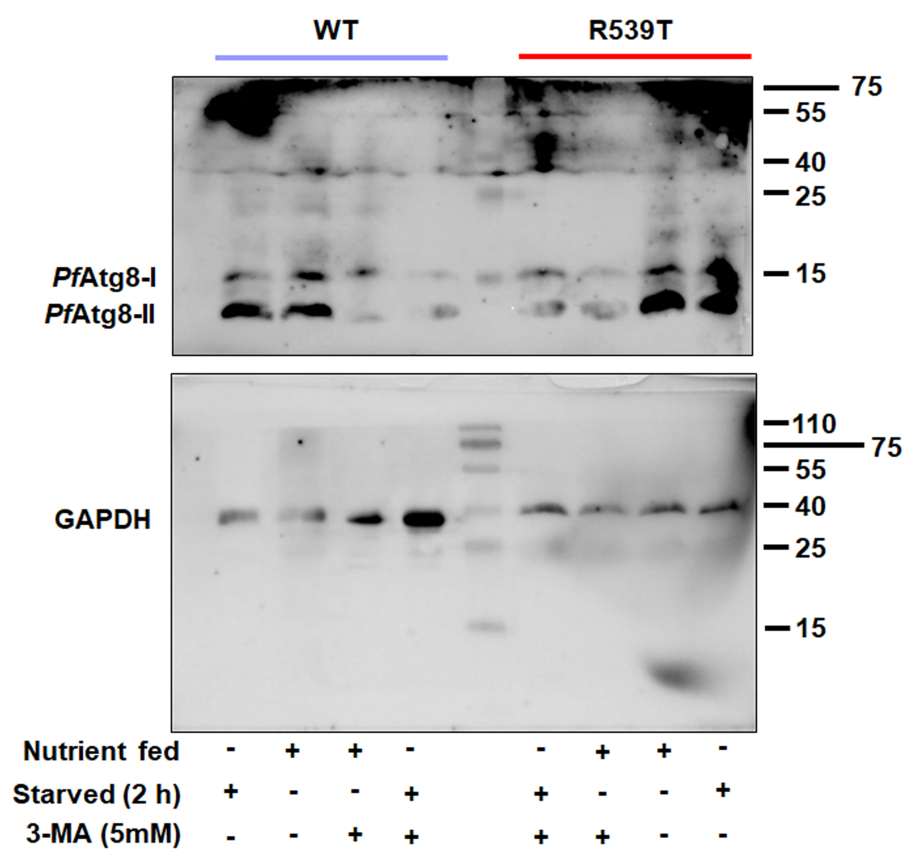

Supplement: Supplementary file 4 — Supplementary Figure 3 [file 41420_2023_1401_MOESM4_ESM.pdf]

**RBC**

**rAtg8**

**M**

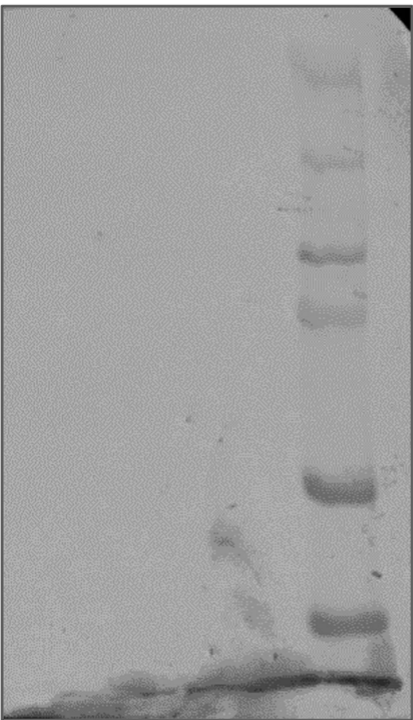

**← 13**

Supplement: Supplementary file 5 — Supplementary Figure 4 [file 41420_2023_1401_MOESM5_ESM.pdf]
